# Relative contribution of IL-1α, IL-1β and TNF to the host response to *Mycobacterium tuberculosis* and attenuated *M. bovis BCG*

**DOI:** 10.1002/iid3.9

**Published:** 2013-10-30

**Authors:** Marie-Laure Bourigault, Noria Segueni, Stéphanie Rose, Nathalie Court, Rachel Vacher, Virginie Vasseur, François Erard, Marc Le Bert, Irene Garcia, Yoichiro Iwakura, Muazzam Jacobs, Bernhard Ryffel, Valerie F J Quesniaux

**Affiliations:** 1 CNRS, UMR7355Orleans, France; 2 University of Orleans, Experimental and Molecular Immunology and NeurogeneticsOrleans, France; 3 Department of Pathology and Immunology, University of Geneva Medical SchoolGeneva, Switzerland; 4 Center for Experimental Medicine, The Institute of Medical Science, University of TokyoTokyo, Japan; 5 Division of Immunology, Institute of Infectious Disease and Molecular Medicine, Health Sciences Faculty, University of Cape TownCape Town, South Africa; 6 National Health Laboratory ServiceCape Town, South Africa

**Keywords:** Host response, IL-1β/IL-1α, *M. bovis* infection, *M. tuberculosis*, TNF

## Abstract

TNF and IL-1 are major mediators involved in severe inflammatory diseases against which therapeutic neutralizing antibodies are developed. However, both TNF and IL-1 receptor pathways are essential for the control of *Mycobacterium tuberculosis* infection, and it is critical to assess the respective role of IL-1α, IL-1β, and TNF. Using gene-targeted mice we show that absence of both IL-1α and IL-1β recapitulates the uncontrolled *M. tuberculosis* infection with increased bacterial burden, exacerbated lung inflammation, high IFNγ, reduced IL-23 p19 and rapid death seen in IL-1R1-deficient mice. However, presence of either IL-1α or IL-1β in single-deficient mice is sufficient to control acute *M. tuberculosis* infection, with restrained bacterial burden and lung pathology, in conditions where TNF deficient mice succumbed within 4 weeks with overwhelming infection. Systemic infection by attenuated *M. bovis* BCG was controlled in the absence of functional IL-1 pathway, but not in the absence of TNF. Therefore, although both IL-1α and IL-1β are required for a full host response to virulent *M. tuberculosis*, the presence of either IL-1α or IL-1β allows some control of acute *M. tuberculosis* infection, and IL-1 pathway is dispensable for controlling *M. bovis* BCG acute infection. This is in sharp contrast with TNF, which is essential for host response to both attenuated and virulent mycobacteria and may have implications for anti-inflammatory therapy with IL-1β neutralizing antibodies.

## Introduction

Tuberculosis (TB) is still a major health problem, with about one-third of the global population considered to be infected with *Mycobacterium tuberculosis*. Only 5–10% of infected individuals develop an active disease, suggesting that the host immune system is usually efficiently dealing with the mycobacteria, although the infection is not cleared and can remain in a latent form for many years [1,2]. Quantification of bacterial growth and death rates showed that *M. tuberculosis* replicates throughout the course of chronic tuberculosis infection in mice and is restrained by the host immune system [3]. Thus, immunodepression of the host can favour a reactivation of latent tuberculosis infection. Neutralisation of TNF for the treatment of severe inflammatory diseases has been associated with reactivation of latent tuberculosis and increased susceptibility to primary tuberculosis infection [4–7], a risk that is still present when patients do not receive appropriate chemoprophylactic treatment [8]. Although inhibiting IL-1 represents an interesting alternative to TNF neutralisation in severe inflammatory diseases [9], IL-1/IL-1R1 pathway seems also essential for the control of acute *M. tuberculosis* infection [10–16]. Indeed, coordinated innate and adaptive immune responses are required for efficient control of *M. tuberculosis* infection, including T cells, macrophages, and the expression of IFN-γ, TNF, IL-1, IL-12, IL-17A, nitric oxide (NO), reactive oxygen and reactive nitrogen intermediates [17–21].

Isolated cases of pulmonary tuberculosis reactivation in patients treated with short-lived IL-1Ra were reported [22,23]. At a time when antibodies neutralizing IL-1 or IL-1R with long biological half-life are being developed for clinical therapies [9,24–26], it is essential to understand the relative contribution of IL-1α and IL-1β, and to compare it to that of TNF, for the control of *M. tuberculosis* infection. Early studies showed the protective role of the IL-1α/β pathway in *M. tuberculosis* infection using double deficient mice [12] or mice deficient for IL-1R1 [11,13]. An association between IL-1β and the resistance to tuberculosis was inferred from human IL-1B gene polymorphism [27–29]. The role of IL-1β was shown again recently [15], although after immunisation with virus-like particles, neutralisation of IL-1α rather than IL-1β increased mice susceptibility to *M. tuberculosis* [30]. The recent advances in deciphering the inflammasomes and the mechanisms of IL-1α versus IL-1β maturation were calling for a reassessment of the respective role of IL-1α versus IL-1β. Intriguingly, the main inflammasome pathways involved in IL-1β maturation seem dispensable for IL-1β maturation during *in vivo M. tuberculosis* infection, although they contribute *in vitro* [15,31–34]. We were interested in determining the relative contribution of IL-1α and IL-1β in the response to *M. tuberculosis*. An elegant study by Mayer-Barber [16] showed myeloid cell populations co-expressing both IL-1α and IL-1β that are regulated by Type I and Type 2 interferons. However, cross-regulatory mechanisms for release of IL-1α and IL-1β have been reported, with mutual induction of IL-1α and IL-1β, leading to the reduction of IL-1β in the absence of IL-1α, and reduction of IL-1α secretion in the absence of IL-1β acting as a “shuttle” [35–37]. Further, IL-1β and downstream TNF production leading to caspase-dependent restriction of intracellular *M. tuberculosis* growth was recently shown in macrophages *in vitro* [38].

Here we address the relative contribution of IL-1α versus IL-1β in the dramatic impairment of host response to acute *M. tuberculosis* infection seen in the absence of IL-1R1, and compare it to the well established susceptibility of TNF deficient mice [17,18,39–43], using mice deficient for different members of the IL-1/IL-1R1 family, or for TNF, side by side in an acute model of aerogenic *M. tuberculosis* infection. We confirm that both TNF and IL-1 pathways are required to control *M. tuberculosis* infection since absence of both IL-1α and IL-1β recapitulated the dramatic defect seen in the absence of IL-1R1 or TNF. However, presence of either IL-1α or IL-1β allows some control of acute *M. tuberculosis* infection while double deficient mice succumb rapidly. Further, although TNF is essential for the early control of infection by either virulent or attenuated mycobacteria, IL-1 pathway is dispensable for controlling less virulent infection by *M. bovis* BCG.

## Results

### Cross-regulation of IL-1α and IL-1β release by macrophages in response to mycobacteria

**Figure 1:**
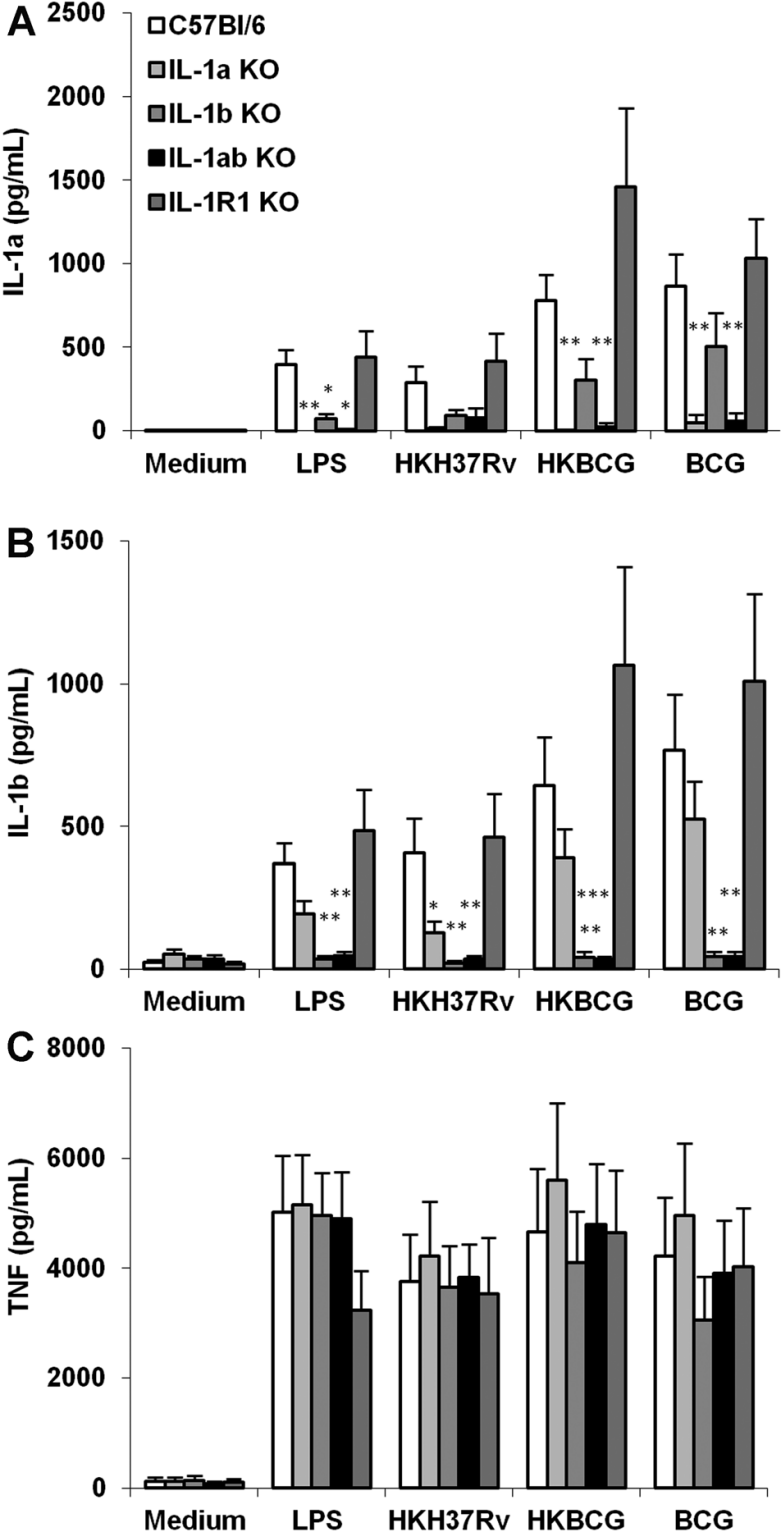
IL-1α and IL-1β cross-regulation in IL-1α and/or IL-1β deficient macrophages in response to mycobacteria. BM derived macrophages prepared from IL-1α and/or IL-1β deficient, IL-1R1 deficient and wild-type mice were incubated with LPS (100 ng/ml), heat-killed *M. tuberculosis* H37Rv (HKH37Rv) or *M. bovis* BCG (HKBCG), or live BCG (BCG), at a MOI of 2. After 24 h, the production of IL-1α (A), IL-1β (B) or TNF (C) was determined in the supernatants by ELISA. Data are mean ± SEM, with *n* = 7 mice per group from three independent experiments (*P < 0.05; **P < 0.01; ***P < 0.001, for each group as compared to the respective wild-type controls).

Since a mutual induction of IL-1α and IL-1β was reported after stimulation with LPS or turpentine [35], and IL-1β has been identified as a necessary “shuttle” for IL-1α secretion after LPS stimulation [37], we first assessed the interdependence of IL-1α and IL-1β expression in response to mycobacteria *in vitro*. Bone marrow derived macrophages from mice deficient singly for IL-1α or IL-1β, for both IL-1α and IL-1β, or for IL-1R1 were stimulated with *M. tuberculosis* H37Rv or *M. bovis* BCG *in vitro* and their ability to secrete IL-1α and IL-1β, or TNF, determined. As expected, no IL-1α was detected in the IL-1α deficient mice (Fig. 1A) and no IL-1β was detected in the IL-1β deficient mice (Fig. 1B). The level of IL-1α was partially reduced in IL-1β deficient macrophages stimulated with TLR4 agonist LPS, *M. tuberculosis* H37Rv or *M. bovis* BCG, although this did not reach statistical significance. Similarly, the level of IL-1β was slightly reduced in the IL-1α deficient macrophages stimulated with LPS, *M. tuberculosis* H37Rv or *M. bovis* BCG. A mutual reduction of IL-1α in the absence of IL-1β and, conversely, a reduction of IL-1β in the absence of IL-1α, were also seen with stimuli triggering different inflammasome pathways such a uric acid crystals (Ref. [36] and unpublished data). Such interdependence of IL-1α and IL-1β release could be due to indirect IL-1 amplification loops, or to the requirement for IL-1β shuttle effect. However, the absence of functional IL-1R1 had little effect on the release of either IL-1α or IL-1β (Fig. 1A and B), pointing against an IL-1/IL-1R1 mediated indirect amplification loop. TNF release induced by mycobacteria was independent of the IL-1α/β/IL-1R1 axis *in vitro* (Fig. 1C), as expected [10–16]. Conversely, the release of IL-1α and IL-1β in response to mycobacteria was not affected in TNF deficient macrophages (data not shown). Thus, there was some interdependence of IL-1α and IL-1β release after macrophage stimulation with TLR agonists or mycobacteria, which did not affect TNF release.

### Lethal *M*. *tuberculosis* infection in the absence of both IL-1α and IL-1β, or TNF pathways

**Figure 2:**
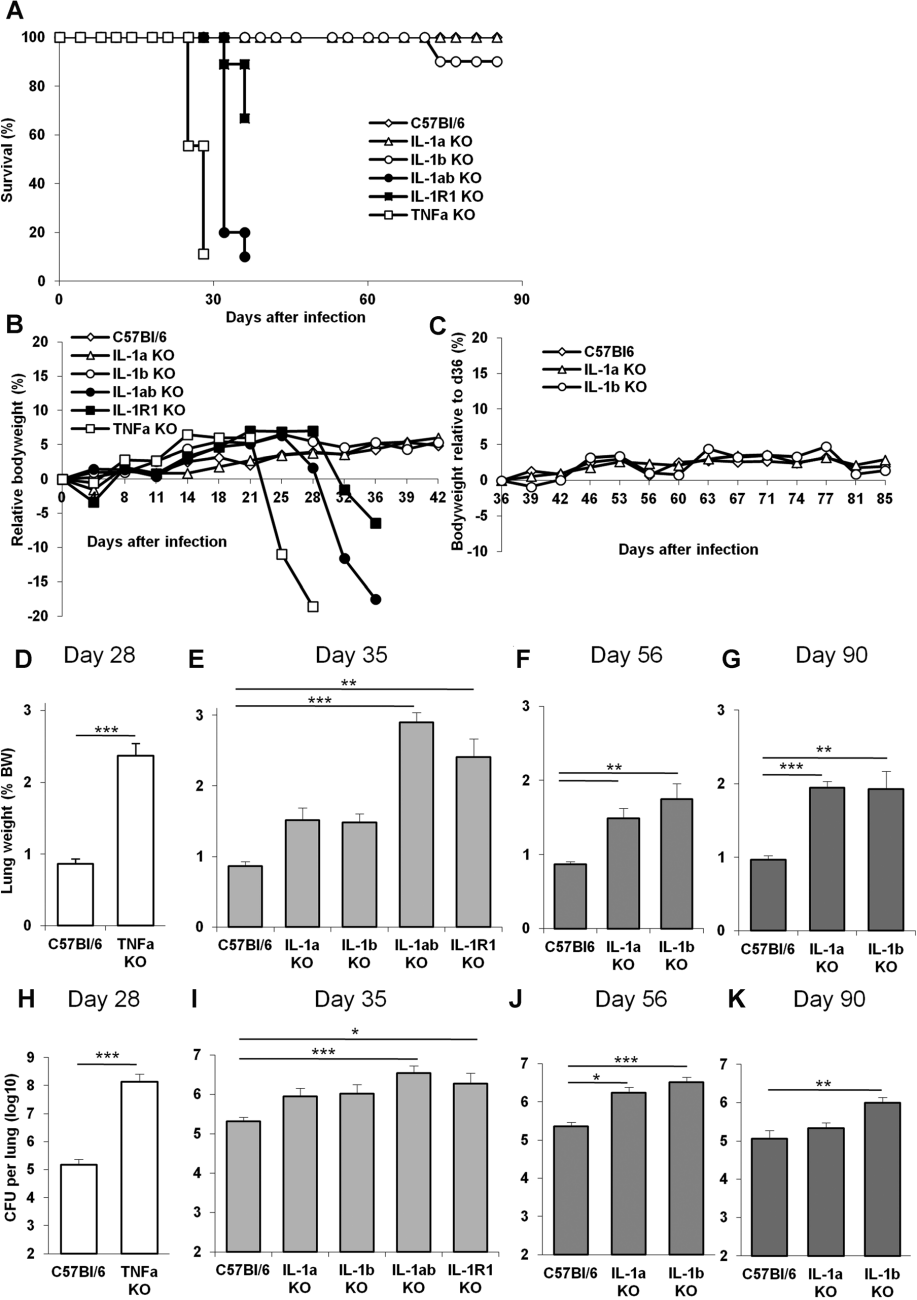
Lethal *M. tuberculosis* infection in IL-1α plus IL-1β double deficient mice is partially controlled in IL-1α or IL-1β single deficient mice. A–C: Mice deficient for IL-1α, IL-1β, IL-1α plus IL-1β, IL-1R1 or TNF and wild-type C57Bl/6 mice were exposed to *M. tuberculosis* H37Rv (1600 ± 300 CFU/mouse i.n.) and monitored for survival (A), bodyweight during acute (B) and more chronic (C) infection. Data are from two representative experiments out of three independent experiments (A: *n* = 10–13 mice/group; B: *n* = 9–11 mice/group for TNF, IL-1R1 and IL-1α plus IL-1β KO mice, 24 for IL-1α and 20 for IL-1β KO mice/group; C: *n* = 15–23 up to day 55 and 8–12 thereafter). D–G: Lung weight of highly sensitive TNF deficient mice is shown on day 28 post-infection when lung inflammation is exacerbated (D), and on day 35 for IL-1α, IL-1β, IL-1α plus IL-1β, IL-1R1 deficient or wild-type mice (E). Lung weight in IL-1α or IL-1β deficient mice surviving the infection on day 56 (F) and day 90 (G) post-infection. H–K: Pulmonary bacterial loads were measured on day 28 for highly sensitive TNF deficient mice (H) and on day 35 post-infection for IL-1α, IL-1β, IL-1α plus IL-1β, IL-1R1 deficient or wild-type mice (I). Lung bacterial loads in IL-1α or IL-1β deficient mice were further assessed on day 56 (J) and day 90 (K) post-infection. Results in D–K are expressed as mean ± SEM of *n* = 8–13 mice pooled from two representative experiments out of four independent experiments (*P < 0.05; **P < 0.01; ***P < 0.001, as compared to wild-type control).

We showed earlier that IL-1R1 deficient mice are extremely sensitive to virulent *M. tuberculosis* H37Rv infection, similar to TNF or MyD88 deficient mice [13,44], and we next wanted to assess the relative contribution of IL-1α and IL-1β to the IL-1R1 mediated immune response to acute *M. tuberculosis* infection. Indeed, among the early studies addressing the role of IL-1 in host response to *M. tuberculosis* [10–12], a direct comparison of mice deficient in IL-1α or IL-1β, both IL-1α and IL-1β, IL-1R1 or TNF was missing. Mayer-Barber et al. [16] recently reported a rapid mortality of mice singly or double deficient for IL-1α and/or IL-1β, within 30–40 days post *M. tuberculosis* infection, although after low dose infection IL-1α or IL-1β single deficient mice succumbed 3–5 months after IL-1R1-deficient mice. Here, mice singly deficient for IL-1α or IL-1β survived the acute phase of infection without bodyweight loss, while mice deficient for both IL-1α and IL-1β, similar to IL-1R1 deficient mice, rapidly lost weight and succumbed within 5 weeks of infection with *M. tuberculosis* H37Rv, that is, 1 week after TNF deficient mice (Fig. 2A,B). However, mice singly deficient for either IL-1α or IL-1β survived for the duration of the acute *M. tuberculosis* infection study (3 months) without clinical symptoms of infection (Fig. 2A-C). Therefore, presence of either IL-1α or IL-1β allows some control of acute *M. tuberculosis* infection.

### Exacerbated pulmonary inflammation after *M*. *tuberculosis* infection in the absence of IL-1α, IL-1β or IL-1R1, alike in the absence of TNF

We and others reported earlier a dramatic increase in lung inflammation in IL-1R1, MyD88 or TNF deficient mice early after *M. tuberculosis* infection [10,11,13,43,44]. Since there seems to be little involvement of the major NLRP3 inflammasome pathway leading to IL-1β maturation [31,32], we wished to address further the relative contribution of IL-1α versus IL-1β in the lung inflammation induced during acute *M. tuberculosis* infection. IL-1α and IL-1β double deficient mice exhibited strongly increased lung weights, an indicator of lung inflammation, 5 weeks post-infection, as did IL-1R1 deficient mice (Fig. 2E) and TNF KO mice which succumbed at 4 weeks (Fig. 2D). Mice deficient only for IL-1α or IL-1β had less pronounced pulmonary inflammation on day 35 post-infection (Fig. 2E). However, IL-1α or IL-1β deficient mice survived to a more chronic infection and lung inflammation was gradually increased in these animals 2 or 3 months post-infection (Fig. 2F and G). Therefore it seemed that expression of either IL-1α or IL-1β helped controlling acute lung inflammation induced by *M. tuberculosis* infection.

As this may be a consequence of a better contained infection, we next determined the bacterial loads in these animals. Mice double deficient for IL-1α plus IL-1β could not control the infection, similar to IL-1R1 deficient mice on day 35 post-infection (Fig. 2I), although bacterial burdens were not as high as those reached in TNF KO mice which succumbed at 4 weeks (Fig. 2H). Deficiency in either IL-1α or IL-1β allowed some bacterial growth restriction on day 35 post-infection (Fig. 2I), and there was a limited increase in bacterial load at 2–3 months post-infection (Fig. 2J and K). Therefore, mice double deficient in IL-1α plus IL-1β develop a rapid, exacerbated lung inflammation which may be the result of uncontrolled bacterial infection, as seen in IL-1R1 or TNF deficient mice, but expression of either IL-1α or IL-1β was sufficient to contribute to the host control of bacterial growth and lung inflammation during acute *M. tuberculosis* infection.

### Presence of either IL-1β or IL-1α prevents the acute necrotic pneumonia seen after *M. tuberculosis* infection in IL-1 pathway defective mice, but do not compensate for lack of TNF

**Figure 3:**
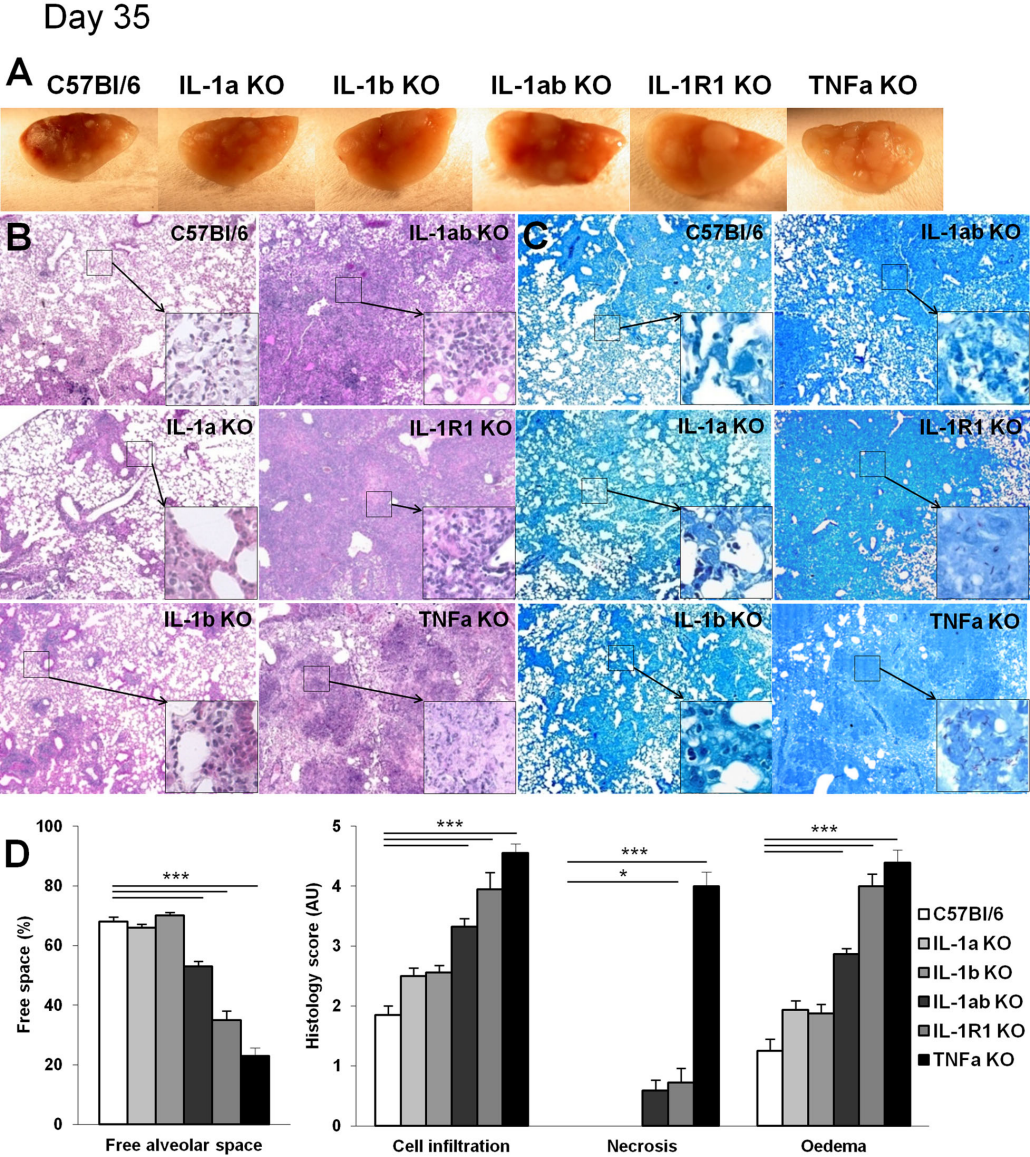
Presence of IL-1α or IL-1β prevents acute necrotic pneumonia in response to *M. tuberculosis* infection. Mice deficient for IL-1α, IL-1β, IL-1α plus IL-1β, IL-1R1 or TNF and wild-type C57Bl/6 mice were exposed to *M. tuberculosis* H37Rv as in Figure 2 and macroscopic lung pathology assessed on day 35. Macroscopically, lungs of IL-1α plus IL-1β deficient mice showed large nodules similar to IL-1R1 deficient lungs (A). Lungs of TNF deficient mice with large, confluent nodules on day 28 post-infection are included for comparison. Microscopic examination showing extensive inflammation and necrosis in infected IL-1α plus IL-1β, and in IL-1R1 deficient lungs (B; Hematoxylin and Eosin, magnification 50× for low power and 200× for details) with abundant mycobacteria in the extracellular space (C; Ziehl-Neelsen, magnification 50× for low power and 1000× for details). Bar graphs (D) summarise free alveolar space and scores of cell infiltration, necrosis and oedema at this time point (*n* = 8–11 mice per group from two independent experiments; *P < 0.05; **P < 0.01; ***P < 0.001, as compared to wild-type control).

**Figure 4:**
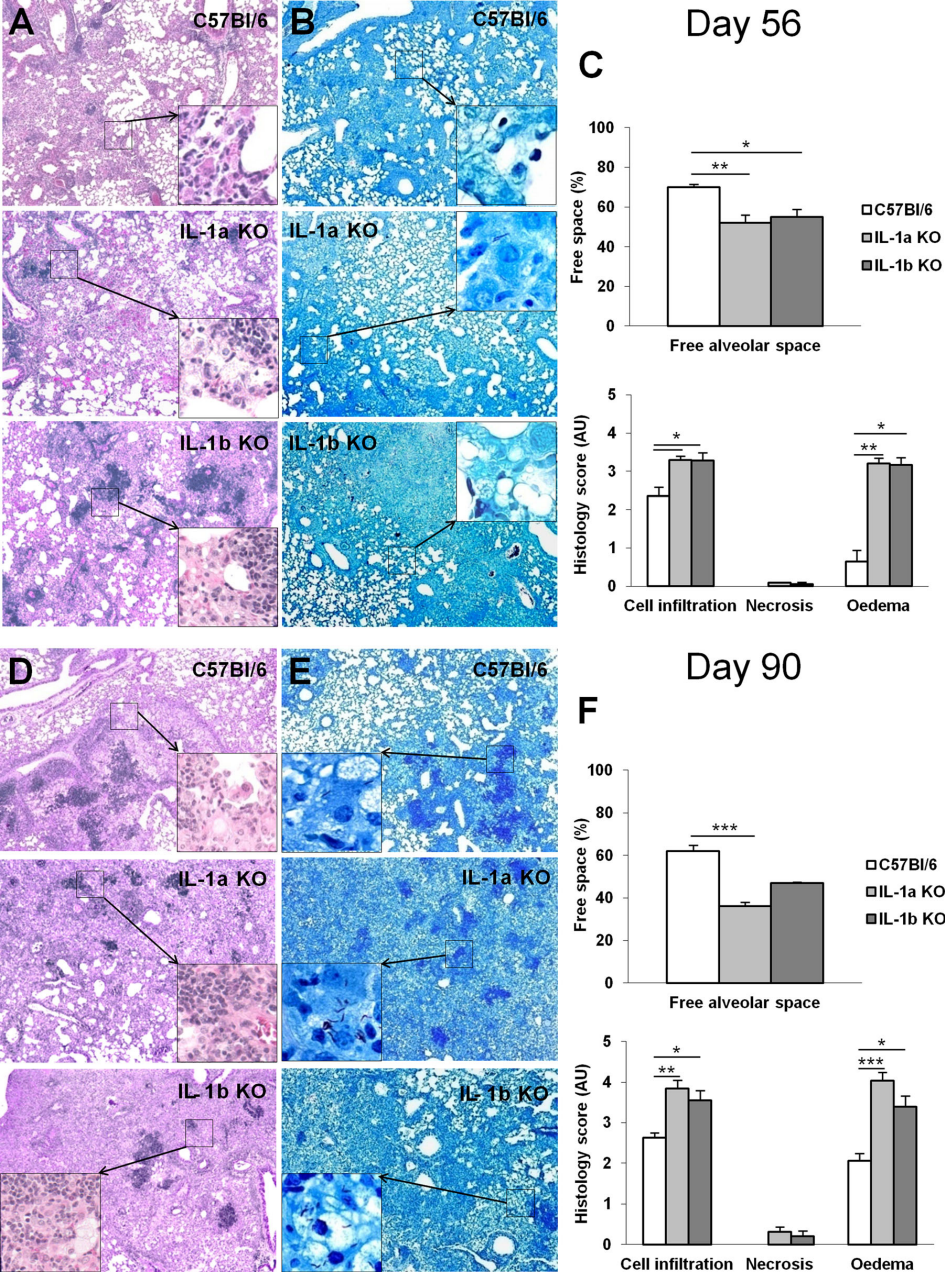
Progressive pneumonia after 2–3 months *M. tuberculosis* infection in the absence of IL-1α or IL-1β. Mice deficient for IL-1α or IL-1β and wild-type C57Bl/6 mice exposed to *M. tuberculosis* H37Rv as in Figure 2 were followed for 56 (A–C) and 90 (D–F) days and lung pathology assessed. Microscopic examination showing progressive inflammation in infected IL-1α or IL-1β deficient lungs (A, D; Hematoxylin and Eosin, magnification 50× for low power and 200× for details) with abundant mycobacteria in the extracellular space (B, E; Ziehl–Neelsen, magnification 50× for low power and 1000× for details). Bar graphs (C, F) summarise free alveolar space and scores of cell infiltration, necrosis, and oedema at these time points (*n* = 8–11 mice per group from two independent experiments; *P < 0.05; **P < 0.01; ***P < 0.001, as compared to wild-type control).

The establishment of well-defined granuloma, result of a structured cell mediated immune response, is thought to be crucial for inhibiting mycobacteria growth. In view of the increased lung weight indicative of a strong local inflammation in mice lacking IL-1α and/or IL-1β, and of the defective granuloma formation reported in IL-1R1 deficient mice [13], we next addressed the relative contribution of IL-1α and IL-1β in granuloma formation upon *M. tuberculosis* infection. Macroscopically, the lungs of mice deficient for both IL-1α and IL-1β displayed pleural adhesions, large confluent nodules, similar to IL-1R1 or TNF deficient mice (Fig. 3A). Microscopic investigation of the lungs of IL-1α plus IL-1β deficient mice revealed severe inflammation with important reduction of ventilated alveolar spaces, massive mononuclear cell and neutrophil infiltrations with extensive confluent necrosis and oedema, in the absence of proper granuloma formation at 35 days (Fig. 3B) and abundant mycobacteria within macrophages and in the extra-cellular space (Fig. 3C). The lung lesions observed in the absence of both IL-1α and IL-1β were similar to those seen in mice deficient for IL-1R1 or TNF, although confluent necrosis was more pronounced in the absence of TNF 4 weeks post-infection (Fig. 3B). A strong iNOS staining confined to macrophages residing in well defined granuloma was seen in wild-type mice on day 35 post-infection (Fig. S1). Pulmonary iNOS expression was attenuated in IL-1α or IL-1β single deficient mice, while in IL-1α and IL-1β double deficient mice and IL-1R1 deficient mice there was a diffuse expression through the inflamed lung at that stage, without strong cell-associated staining as in wild-type mice. Interestingly, mice singly deficient for IL-1α or for IL-1β developed some granuloma with limited oedema, no necrosis and free alveolar space, thus a much less severe lung pathology than that seen in the absence of both IL-1α and IL-1β or of IL-1R1 at 5 weeks post-infection. At 2–3 months post-infection lung pathology in mice deficient for IL-1α or for IL-1β progressed with reduced free alveolar space, increased inflammatory cell infiltration, acid-fast bacilli abundance and oedema, as compared to wild-type mice, but still little necrosis (Fig. 4A and B). Thus, absence of both IL-1α and IL-1β recapitulates the acute necrotic pneumonia resulting from defective IL-1R1 or TNF pathways, while the sole presence of IL-1α or IL-1β limits but does not fully prevent lung infiltration of inflammatory cells, oedema and necrosis after acute *M. tuberculosis* infection.

### Regulation of pulmonary cytokine expression by IL-1α/IL-1β pathway after *M. tuberculosis* infection

**Figure 5:**
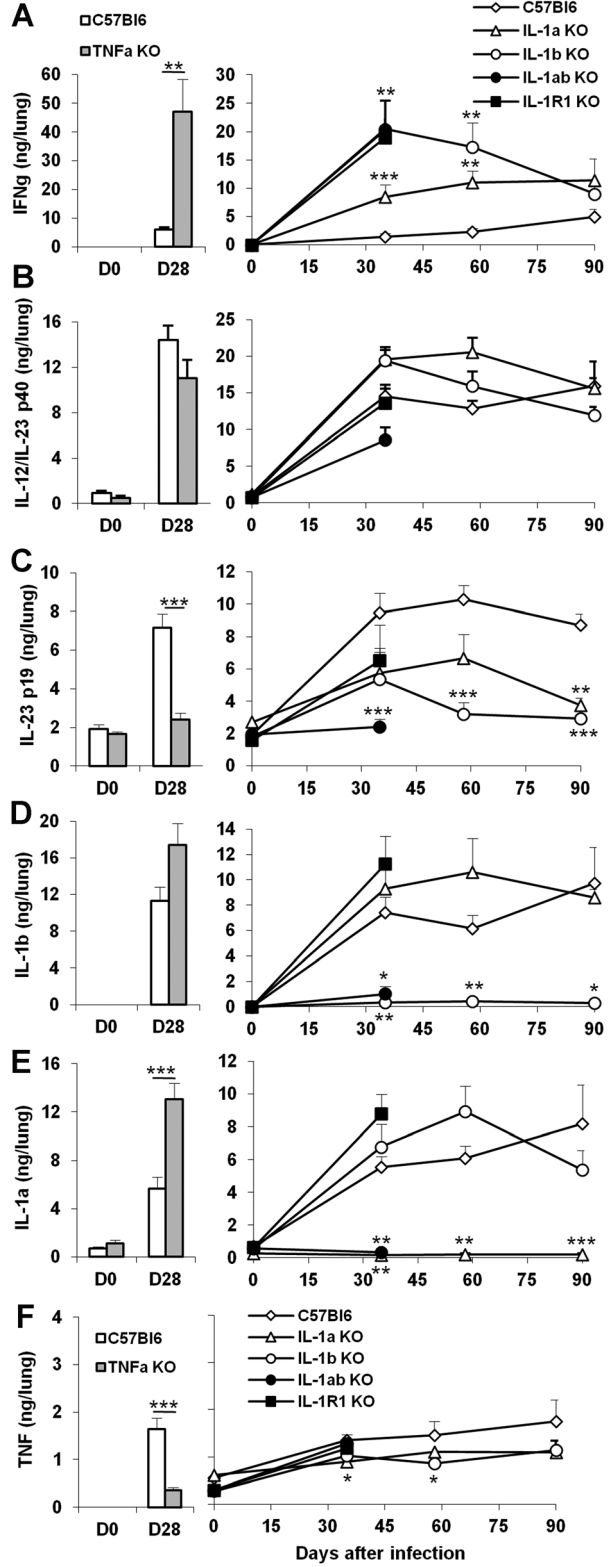
Cytokine pulmonary levels in *M. tuberculosis* infected TNF or IL-1 deficient mice. Cytokine concentrations were determined in lung homogenates of TNF deficient and wild-type mice 28 days after *M. tuberculosis* infection (left panels). Cytokine lung levels of mice deficient for IL-1α, IL-1β, IL-1α plus IL-1β, or IL-1R1 and wild-type mice are shown at 0, 35, 56, and 90 days after *M. tuberculosis* infection (right panels). IFNγ (A), IL-12/IL-23 p40 (B), IL-23 p19 (C), IL-1β (D), IL-1α (E) and TNFα (F) were quantified by ELISA. Results are expressed as mean ± SEM of cytokine levels reported to whole lungs, from *n* = 8–11 mice per group from two independent experiments (*P < 0.05; **P < 0.01; ***P < 0.001, as compared to wild-type control at the respective time point).

We next assessed the expression of cytokines involved in innate and adaptive immune responses in these mice. Indeed, the uncontrolled bacilli growth and pulmonary inflammation in mice deficient for both IL-1α and IL-1β was reminiscent of the high infectious burden in *M. tuberculosis* infected IL-1R1 or MyD88 deficient mice, which was accompanied by increased macrophage and neutrophil recruitment, but a normal adaptive response in terms of recruitment, activation and priming of effector T cells [13,44]. Further, in view of the persistent lung inflammation in single IL-1α or IL-1β deficient mice during chronic *M. tuberculosis* infection, we asked how the absence of IL-1α and/or IL-1β modulated the immune response in the lung after 1–3 months of infection (Fig. 5). Lung IFNγ levels were highly elevated in mice deficient for both IL-1α and IL-1β or for IL-1R1 at 5 weeks after *M. tuberculosis* infection (Fig. 5A), reminiscent of TNF deficient mice at 4 weeks, when they succumb to infection. Interestingly, IFNγ levels were also highly elevated in IL-1β deficient mice, but less so in IL-1α deficient mice, indicating that presence of IL-1β sufficed to control IFNγ over-expression. While IL-12/23 p40 levels were reduced in mice deficient for both IL-1α and IL-1β (Fig. 5B), they were essentially normal in IL-1R1 deficient mice or TNF deficient mice and slightly elevated in mice deficient for either IL-1α or IL-1β, although this did not reach statistical significance. IL-23 p19 pulmonary levels were reduced in the highly susceptible, TNF-deficient mice at 4 weeks of *M. tuberculosis* infection and IL-23 p19 levels were also clearly lower than wild-type controls in mice deficient for both IL-1α and IL-1β (Fig. 5C), and partially decreased in IL-1R1 deficient mice or mice deficient for either IL-1α or IL-1β, 5 weeks after *M. tuberculosis* infection. The levels of IL-17A were also reduced in mice deficient for both IL-1α and/or IL-1β or IL-1R1 at 5 weeks post-infection and in IL-1α or IL-1β single deficient mice at 8 weeks post-infection (Fig. S2). IL-1α and/or IL-1β gene deficient mice did not express IL-1α and/or IL-1β as expected, while IL-1α and IL-1β pulmonary levels were elevated in TNF-deficient mice at 4 weeks of *M. tuberculosis* infection, and were also elevated in IL-1R1 deficient mice as compared to wild-type control 5 weeks post infection. IL-1α levels were slightly increased in IL-1β deficient mice, and conversely, IL-1β levels were slightly increased in IL-1α deficient mice during the first 2 months of infection (Fig. 5D and E). TNF pulmonary levels were rather modest, as shown previously [44] and were partially reduced in the absence of IL-1α and/or IL-1β, or IL-1R1 (Fig. 5F). Therefore, complete absence of IL-1α plus IL-1β led to increased IFNγ pulmonary levels, but decreased IL-12/23 p40 and IL-23 p19 expression, which were partially corrected by the presence of either IL-1α or IL-1β. In line with TNF being downstream of IL-1β for restricting *M. tuberculosis* growth in macrophages *in vitro* [38], absence of IL-1 pathway impacted on partial reduction of TNF pulmonary levels after *in vivo M. tuberculosis* infection, while they was no reduction of IL-1α or IL-1β lung content in the absence of TNF.

### IL-1 pathway is dispensable for controlling attenuated *M. bovis* BCG infection, while TNF is essential

Mice deficient for TNF are highly susceptible not only to virulent *M. tuberculosis*, but also to attenuated *M. bovis* BCG (Refs. [39–41,45–47] and unpublished data). In contrast, mice deficient for MyD88, an essential adaptor of IL-1R1 pathway, were reported as highly susceptible to virulent *M. tuberculosis* infection by us and others [13,48,49], while they controlled chronic *M. bovis* BCG infection [50] and were found less susceptible to *M. tuberculosis* by other authors [51]. Our data indicated that although IL-1R1 pathway is essential for controlling acute infection by virulent *M. tuberculosis*, presence of either IL-1α or IL-1β allowed to survive acute *M. tuberculosis* infection. In previous reports, deficiency in IL-1α or IL-1β led to either early death after *M. tuberculosis* infection or survival up to 3–6 months at lower dose infection [16]. We next asked how the complete absence of IL-1 pathway affects the control of less virulent mycobacteria.

**Figure 6:**
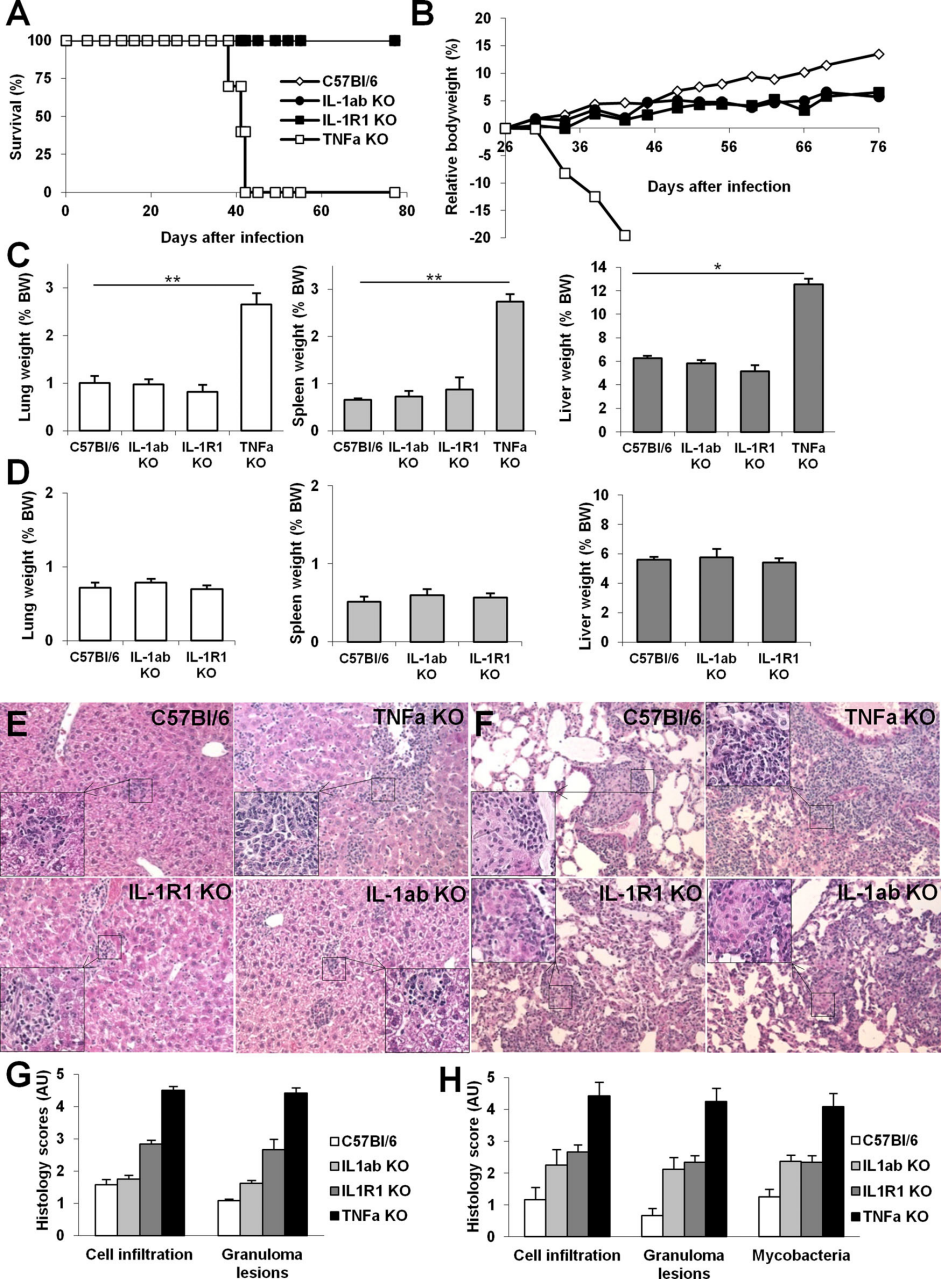
TNF is crucial, but IL-1α/IL-1β pathway is dispensable for controlling *M. bovis* BCG infection. Mice deficient for IL-1α plus IL-1β, IL-1R1 or TNF and wild-type C57Bl/6 mice were infected with *M. bovis* BCG (10^6^ CFU/mouse, iv) and monitored for survival (A) and bodyweight (B). Lung, spleen and liver weights, surrogate markers of inflammation, were highly increased in sensitive TNF deficient mice, as compared to wild-type mice or mice deficient for IL-1α plus IL-1β or IL-1R1 at 6 weeks (C) or 11 weeks (D) post-infection. Data are pooled from two independent experiments with *n* = 10–15 mice/group up to day 41 and *n* = 7–8 mice/group thereafter (*P < 0.05; **P < 0.01; ***P < 0.001, as compared to wild-type control). Microscopic examination of liver (E) and lung (F) at 6 weeks showed extensive inflammatory cell infiltration, granulomatous lesions and oedema in TNF deficient mice while this was much less pronounced in mice deficient for IL-1α plus IL-1β, or IL-1R1 (Hematoxylin and Eosin, magnification 50× for low power and 200× for details). Bar graphs (G, H) summarise scores of cell infiltration, extent of granulomatous lesions, and presence of visible mycobacteria in liver (G) and lung (H), respectively (*n* = 3–6 mice per group from one experiment representative of two independent experiments).

Mice deficient for IL-1α plus IL-1β, IL-1R1 or TNF were infected with *M. bovis* BCG (Fig. 6). TNF deficient mice rapidly lost weight and succumbed by 6 weeks of infection (Fig. 6A and B) with highly inflammed lung, spleen and liver, while IL-1R1 and IL-1α plus IL-1β deficient mice survived systemic *M. bovis* BCG infection with lung, spleen and liver weight similar to wild-type mice at 6 and 11 weeks (Fig. 6C and D). Histologically, liver and lung inflammation were exacerbated in TNF deficient mice, with numerous and large, confluent granulomatous structures containing massive cell infiltrates, while the pathology was much less severe in IL-1R1 or IL-1α plus IL-1β deficient mice (Fig. 6E and F). Therefore, the IL-1 pathway which is essential for controlling virulent *M. tuberculosis* infection is dispensable for the early control of attenuated *M. bovis* BCG infection, in sharp contrast with TNF which is essential for both responses.

## Discussion

Therapeutical neutralisation of TNF in the treatment of severe inflammatory diseases has been associated with reactivation of latent tuberculosis and increased susceptibility to primary tuberculosis infection [4–7], and inhibiting IL-1 might represent an interesting alternative [9]. Indeed IL-1, another potent mediator of inflammation involved in the pathogenesis of severe inflammatory and autoimmune diseases such as rheumatoid arthritis, has been neutralised using the IL-1R antagonist Anakinra with good safety record [52], although occasional tuberculosis reactivation have been reported [22,23]. Because of its short half-life, Anakinra needs to be administered daily and it is likely that the neutralisation of IL-1R is not complete, allowing some control of infection. However, new generation antibodies in development for autoimmune or auto-inflammatory disease indications, have much longer half-life, typically 2–3 weeks, and may fully neutralise IL-1β [24–26] or IL-1R1 [9]. We and others showed in mice that the IL-1R pathway is essential for controlling acute tuberculosis infection [10,11,13,14,16]. Therefore, efficient and persistent IL-1 neutralisation with long-lasting anti-IL-1 antibodies may result in reduced host resistance and flare-up of *M. tuberculosis* infection. Since IL-1α might compensate for IL-1β in host response to *M. tuberculosis*, it is critical to determine the respective role and potential redundancy of IL-1α and IL-1β in this response.

The relative contribution of IL-1α and IL-1β in the control of *M. tuberculosis* infection is however still unset. Indeed, a role for IL-1β in tuberculosis was inferred from human IL-1B gene polymorphism [27–29] and experimental studies in IL-1β deficient mice [15], although specific immunisation with virus-like particles leading to neutralisation of IL-1α was shown to increase mice susceptibility to *M. tuberculosis*, while neutralisation of IL-1β did not [30]. Mayer-Barber et al. [16] showed recently that both IL-1α and IL-1β are critically required for host resistance and that absence of either IL-1α or IL-1β severely compromised host response to *M. tuberculosis*, although IL-1 single deficient mice succumb 3–5 months after IL-1R1 deficient mice at low dose infection. Here, we further analysed the respective roles of IL-1α and IL-1β, as compared with TNF in host response to infection by virulent *M. tuberculosis* but also attenuated *M. bovis* BCG. We show that absence of both IL-1α plus IL-1β recapitulated the overwhelming infection and acute necrotic pneumonia seen in IL-1R1 deficient mice, while presence of either IL-1α or IL-1β allowed to control acute *M. tuberculosis* infection and pulmonary inflammation, with limited lung infiltration of inflammatory cells, oedema and necrosis. This may be attributed to a purely quantitative effect, or to non-redundant functions of the individual cytokines that have additive or synergistic effects. However, the compensation of IL-1α by IL-1β, and conversely, was not complete since absence of one IL-1 led to development of increased pathology as compared to wild-type mice, even in the presence of the other IL-1. We thus propose that the presence of IL-1α or IL-1β is sufficient to trigger IL-1R pathway, an essential component in the development of innate response to acute *M. tuberculosis* infection, but may not be sufficient for full control of the infection.

Inter-dependence of IL-1α and IL-1β expression and/or release was long recognised [35], and recently ascribed to IL-1β acting as a shuttle for secretion of mature IL-1α [37]. Here, we show that bone marrow-derived macrophages deficient for IL-1α are somewhat defective in IL-1β production *in vitro*, and conversely, that macrophages deficient for IL-1α exhibit a reduction in IL-1β release in response to mycobacterial antigen stimulation. This reduction was less seen after infection with live *M. bovis* BCG. A difference in the TLR usage of heat-killed versus viable *M. bovis* BCG was reported earlier [53], indicating that live mycobacteria activate other pathways [54]. An indirect IL-1 amplification loop is unlikely since absence of functional IL-1R1 did not hamper the release of IL-1α or IL-1β. The inter-dependence of IL-1α and IL-1β expression or release is more difficult to assess *in vivo*, where cytokine levels are the results of multiple regulations and indirect effector mechanisms. Coproduction of IL-1α and IL-1β at the single cell level was recently documented in distinct myeloid cell populations in the lung of *M. tuberculosis* infected mice [16]. Here, we report slightly increased pulmonary IL-1α levels in IL-1β deficient mice, and conversely, slightly increased pulmonary IL-1β levels in IL-1α deficient mice during acute *M. tuberculosis* infection. This was in line with increased concentrations of IL-1α reported in BAL and lungs of IL-1β deficient mice [15]. In a more recent study, BAL IL-1α levels seemed unaffected in IL-1β deficient mice, and conversely, BAL IL-1β levels were unaffected in IL-1α deficient mice 4 weeks after *M. tuberculosis* infection, while Ly6G^neg^CD11b^pos^ myeloid cells producing IL-1α and/or IL-1β were halved in these mice [16]. We show that absence of both IL-1α and IL-1β led to increased pulmonary levels of IFNγ as reported earlier for IL-1R1-deficient mice [13], in line with the increased IFNγ levels reported in the BAL of these mice 4 weeks post-*M. tuberculosis* infection [16]. The increased IFNγ levels, also seen in the single absence of IL-1α or IL-1β, or of TNF, might be a consequence of the failure of the innate immune response to control the high bacilli burden seen in these mice after acute *M. tuberculosis* infection and were associated with a strong lung inflammation. Indeed, in a model of *M. tuberculosis* infection controlling bacterial burden with streptomycin-dependent strain18b, neutrophil recruitment to the lung was reduced in IL-1R1 deficient mice [21]. However, not all pro-inflammatory, Th1 promoting cytokines were upregulated in the absence of IL-1 or TNF pathways in our model, since there was little effect on IL-12/23 p40 and rather a decrease in IL-23 p19 expression. The slightly increased IL-1β levels in single IL-1α deficient mice were associated with a partial correction of IFNγ and IL-23 p19 levels during acute *M. tuberculosis* infection. Further, IL-17 was strongly reduced in the lung of *M. tuberculosis* infected IL-1 deficient mice, which may contribute to the enhanced susceptibility of these mice.

The high bacterial burden associated with acute *M. tuberculosis* infection in mice lacking either TNF or functional IL-1 pathway may provide abundant PAMPs to trigger cytokine release directly. We showed earlier that mice deficient for MyD88 expressed high levels of IL-1β, TNF, IFNγ and chemokines MCP1 and MIP1α 5 weeks after *M. tuberculosis* infection [44], indicating that TLR, MyD88-dependent pathways were not essential for this strong release of cytokines and chemokines, as confirmed in [15]. Specific PAMPs associated with live bacteria, vitaPAMPs, that are not activating TLR2 and TLR4 but signal through TRIF [54], might play a role during acute, exacerbated *M. tuberculosis* infection. However, high levels of IL-1β and TNF were reported in the BAL and lung of TRIF/MyD88 double deficient mice [15], indicating that neither classical TLR agonists, nor TRIF-dependent vitaPAMPs are essential for the increase in cytokine release seen during acute *M. tuberculosis* infection. A high redundancy between different classes of host pattern recognition receptors susceptible of interacting with mycobacterial motives is likely, including Dectin-1 which induces caspase-1 or non-canonical caspase-8 in response to *M. tuberculosis* or *M. bovis* BCG [55,56]. Clearly, DAMPs, danger associated molecular patterns, released by dying or necrotic cells could contribute to entertain the inflammatory context in highly infected lungs. However, DAMPs activated inflammasomes do not seem essential for *in vivo* host response to *M. tuberculosis*, since mice deficient for NLRP3, ASC or caspase-1 [15,31–34], or for NLRP2, AIM2 or purinergic receptor P2X7 control acute *M. tuberculosis* infection (data not shown). Therefore, while the control by NO of *M. tuberculosis* induced immunopathology and IL-1β production was recently ascribed to NLRP3 thiol nitrosylation and inhibition of NLRP3 inflammasome assembly by NO [21], IL-1β maturation during *M. tuberculosis* infection seems largely independent of the “classical” inflammasomes. Here, we show a reduced expression of iNOS in the lungs of IL-1 defective mice 5 weeks post-infection, which may contribute to their susceptibility to *M. tuberculosis* infection. Molecular mechanisms by which IL-1β directly activates host resistance to *M. tuberculosis* were recently addressed in macrophages *in vitro* [38]. Similar to Tim3, the ligand of Galectin-9 expressed on *M. tuberculosis* infected macrophages, IL-1β inhibited the replication of *M. tuberculosis* in infected macrophages. IL-1β increased TNF release and TNFR1 cell surface expression in *M. tuberculosis* infected macrophages, which led to caspase-3 activation, apoptosis, and restriction of *M. tuberculosis* growth by efferocytosis. In contrast, IL-1α was less effective. IL-1β autocrine action on macrophages is however not believed to be the sole source of TNF *in vivo*, and the antimicrobial effect of TNF was independent of IL-1β [38]. Here, although absence of functional IL-1 pathway had little effect on TNF response to mycobacteria in macrophages *in vitro*, the pulmonary TNF levels were partially reduced *in vivo* in *M. tuberculosis* infected IL-1 deficient mice.

Various results on the importance of IL-1 pathways for controlling mycobacterial infections were obtained using mice with defective IL-1-related genes [10–13]. We hypothesised that the requirement for IL-1 responses depends on the virulence of the mycobacteria. Indeed, although MyD88 deficient mice were reported as highly susceptible to virulent *M. tuberculosis* infection [13,48,49], they control chronic *M. bovis* BCG infection [50], and were found less susceptible to *M. tuberculosis* by others using a different *M. tuberculosis* strain [51]. This was in contrast to TNF deficient mice, which are highly susceptible not only to virulent *M. tuberculosis*, but also to attenuated *M. bovis* BCG [39–41,45–47]. Different regulation of caspase-1 dependent IL-1β release by type I IFNs was reported in response to *M. tuberculosis* or *M. bovis* BCG infection in human macrophages [57]. We thus assessed how mice deficient for IL-1R1, or IL-1α plus IL-1β, could control attenuated mycobacterial infection. We showed that absence of IL-1R1, or IL-1α plus IL-1β, did not compromise the control of a *M. bovis* BCG infection that was fatal for TNF deficient mice within 4–5 weeks. The different results obtained on the importance of IL-1 pathways for controlling mycobacterial infections might thus be at least in part explained by difference in virulence of the mycobacteria strains used. Therefore, the IL-1 pathway is dispensable for the immediate, innate response to control *M. bovis* BCG infection while TNF is essential for the early control of mycobacterial infection even by attenuated *M. bovis* BCG. This is in line with the fact that IL-1β requires TNF pathway to inhibit *M. tuberculosis* replication in infected macrophages, while TNF antimicrobial effects are independent of IL-1β [38]. This may also be related to the multifold source of TNF, the TNF of macrophage/neutrophil origin being crucial for the immediate response, while T-cell derived TNF is important at later stages of the infection [58]. Indeed, mice deficient for TNF expression in macrophages/neutrophils displayed early, transient susceptibility to *M. tuberculosis* but recruited activated TNF-producing T-cells and controlled chronic infection, whereas deficient TNF expression specifically in T-cells resulted in early control but enhanced susceptibility during chronic infection with increased pulmonary pathology, and TNF inactivation in both myeloid and T-cells rendered mice critically susceptible to infection, similar to TNF deficient mice [58]. We propose that TNF from innate, macrophage/neutrophil source is sufficient to keep an infection by attenuated *M. bovis* BCG under control until adaptive response with TNF from T cell origin comes in play. However, to control an acute infection by virulent *M. tuberculosis* the amplification loop provided by IL-1β through upregulation of TNF in infected macrophages *in vitro* [38] and here *in vivo*, seems to be necessary.

Thus, in contrast to TNF, which is essential for the early control of infection by either virulent or attenuated mycobacteria, IL-1 pathway is not central for controlling less virulent mycobacteria such as *M. bovis* BCG. Presence of either IL-1α or IL-1β allows some control of acute *M. tuberculosis* infection. For pharmacological blockade by long-lasting neutralizing monoclonal antibodies it might thus be preferable to target specifically IL-1β, or IL-1α, rather than broadly neutralizing both IL-1α and IL-1β or IL-1R itself, to retain some host immune control of *M. tuberculosis* infection.

## Materials and Methods

### Mice

Mice deficient for IL-1α or IL-1β [35], or both IL-1α and IL-1β [12], IL1-R1 [59], or TNF [60] were bred in the Transgenose Institute animal facility (CNRS UPS44, Orleans). All mice were backcrossed at least 7–10 times on C57BL/6 genetic background. For experiments, adult (8–12 weeks old) animals were kept in isolators in a biohazard animal unit. The infected mice were monitored regularly for clinical status and weighed twice weekly. All animal experiments complied with the French Government's animal experiment regulations and were approved by the “Ethics Committee for Animal Experimentation of CNRS Campus Orleans” (CCO; N°CLE CCO 2011-026).

### Bacteria and infection

*M. tuberculosis* H37Rv (Pasteur) aliquots kept frozen at −80°C were thawed, diluted in sterile saline containing 0.05% Tween 20 and clumping was disrupted by 30 repeated aspirations through a 26 gauge needle (Omnican, Braun, Germany). Pulmonary infection with *M. tuberculosis* H37Rv was performed by delivering 1600 ± 300 CFU/lung into the nasal cavities (20 µl each) under xylazine–ketamine anaesthesia, and the inoculum size was verified 24 h after infection by determining bacterial load in the lungs. Alternatively, *M. bovis* BCG (Pasteur strain 1173P2) or fluorescent GFP-expressing *M. bovis* BCG (gift from Dr. V. Snewin, Wellcome Trust London, UK) was grown to mid-log phase in Middlebrook 7H9 liquid medium (Difco Laboratories, Detroit, MI) supplemented with 10% oleic acid/albumin/dextrose/catalase (OADC, Difco Laboratories) and 0.05% Hygromycin (Invivogen, San Diego, CA) at 37°C, stored at −80°C in 10% glycerol (Sigma, St Louis, MO), and injected intravenously at 10^6^ CFU/mouse.

### Bacterial load in tissues

Bacterial loads in the lung of infected mice were evaluated at different time points after infection with *M. tuberculosis* H37Rv as described [46]. Organs were weighed and defined aliquots were homogenised in PBS in a Dispomix homogeniser (Medic Tools, Axonlab, Baden-Daettwil, Switzerland). Tenfold serial dilutions of organ homogenates in 0.05% Tween 20 containing 0.9% NaCl were plated in duplicates onto Middlebrook 7H11 (Difco) agar plates containing 10% OADC and incubated at 37°C. Colonies were enumerated at 3 weeks and results are expressed as log_10_ CFU per organ.

### Pulmonary cytokine determination

Lung homogenates were centrifuged (3 min at 14,500 rpm), the supernatants sterilised by centrifugation through 0.22 μm filter (3 min at 14,500 rpm; Costar-Corning, Badhoevedorp, The Netherlands), immediately frozen on dry ice and stored at −80°C until determination of IL-1α, IL-1β, IL-12/IL-23p40, IL-23p19, TNF and IFNγ levels by ELISA (Duoset R&D Systems, Abingdon, UK).

### Histopathological analysis

For histological analysis left lobe of lungs from *M. tuberculosis* infected mice were fixed in 4% phosphate buffered formalin and paraffin-embedded. Two to 3-μm sections were stained with Hematoxylin and Eosin and a modified Ziehl-Neelsen method. The latter involved staining in a prewarmed (60°C) carbol-fuchsin solution for 10 min followed by destaining in 20% sulphuric acid and 90% ethanol before counterstaining with methylene blue. Free alveolar space, lung cellular infiltration, oedema and necrosis were quantified using a semi-quantitative score with increasing severity of changes (0–5) by two independent observers including a trained pathologist (BR). Liver and lung from *M. bovis* BCG infected mice were assessed histologically by H&E staining and the slides scored for inflammatory cell infiltration and granuloma lesions as above.

### Primary macrophage cultures

Murine bone marrow cells were isolated from femurs and differentiated into macrophages after culturing at 10^6^ cells/ml for 7 days in DMEM (Sigma) supplemented with 10 mM l-glutamine, 25 mM Hepes, 100 U/ml penicillin and 100 U/ml streptomycin, plus 20% horse serum and 30% L929 cell-conditioned medium as a source of M-CSF [43]. Three days after washing and re-culturing in fresh medium, the cell preparation contained a homogenous population of macrophages. Macrophages were plated in 96 well microculture plates (at 10^5^ cells/well in supplemented DMEM as above), and stimulated with LPS (*Escherichia coli*, serotype O111:B4, Invivogen, Saint Louis, MO at 100 ng/ml), heat-killed *M. tuberculosis* H37Rv (heat-killed 90 min at 80°C; 2 bacteria per cell), live or heat-killed *M*. *bovis* BCG (HKBCG; from Pasteur Institute, Paris, both at a MOI of 2 bacteria per cell). Cell supernatants were harvested after 24 h of stimulation for IL-1α, IL-1β and TNF quantification by ELISA (R&D Duoset).

### Statistical analysis

Statistical significance was determined with Graph Pad Prism (version 5.04 for Windows, GraphPad Software, La Jolla, CA). Differences between multiple in vivo groups were analysed by means of one-way non-parametric ANOVA test (Kruskal–Wallis followed by Dunn's multiple comparison test) and values of *P* ≤ 0.05 were considered significant. Two-tailed, non-parametric Mann–Whitney *t*-test was used for analyzing *in vitro* results in Figure 1.

## SUPPORTING INFORMATION

**Figure S1.** Expression of iNOS in lung tissues of *M. tuberculosis* infected IL-1 deficient mice. Mice deficient for IL-1α, IL-1β, IL-1α plus IL-1β, IL-1R1 or wild-type C57Bl/6 mice were exposed to *M. tuberculosis* H37Rv as in Figure 2 and the expression of iNOS in lung tissue assessed by immunostaining on day 35 post-infection (Magnification 100×; representative sections of *n* = 5 mice per group are shown).

**Figure S2.** Pulmonary levels of IL-17A in *M. tuberculosis* infected IL-1 deficient mice. IL-17A concentrations in lung homogenates of mice deficient for IL-1α, IL-1β, IL-1α plus IL-1β, or IL-1R1 and of wild-type mice were quantified by ELISA (Duoset R&D Systems) at 35 days after *M. tuberculosis* infection (left panel) or 56 days after *M. tuberculosis* infection (right panel). Results are expressed as mean ± SEM of cytokine levels reported to whole lungs, and are from *n* = 5 mice per group, except for two controls at day 56 (**P* < 0.05; ***P* < 0.01; ****P* < 0.001, as compared to wild-type controls).
