# Supplementary figures and images for: Relative contribution of IL-1α, IL-1β and TNF to the host response to Mycobacterium tuberculosis and attenuated M. bovis BCG
Source: Immun Inflamm Dis. 2013 Oct 30;1(1):47–62. doi: 10.1002/iid3.9 (PMC4217540; doi:10.1002/iid3.9)

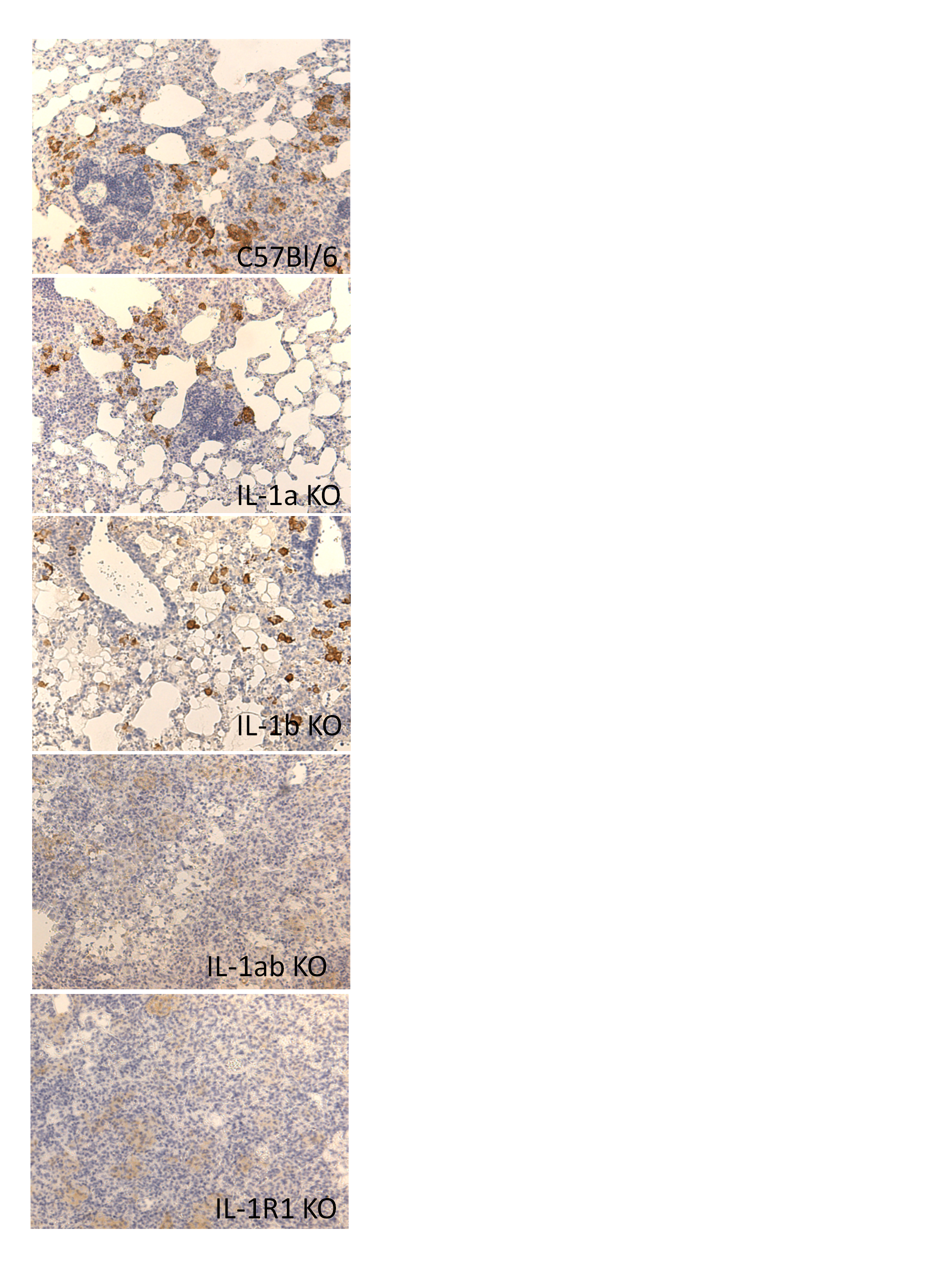

Supplement: Supplementary file 1 — Figure S1. Expression of iNOS in lung tissues of M. tuberculosis infected IL-1 deficient mice. Mice deficient for IL-1α, IL-1β, IL-1α plus IL-1β, IL-1R1 or wild-type C57Bl/6 mice were exposed to M. tuberculosis H37Rv as in Figure 2 and the expression of iNOS in lung tissue assessed by immunostaining on day 35 post-infection (Magnification 100×; representative sections of n = 5 mice per group are shown). [file iid30001-0047-SD1.tif]

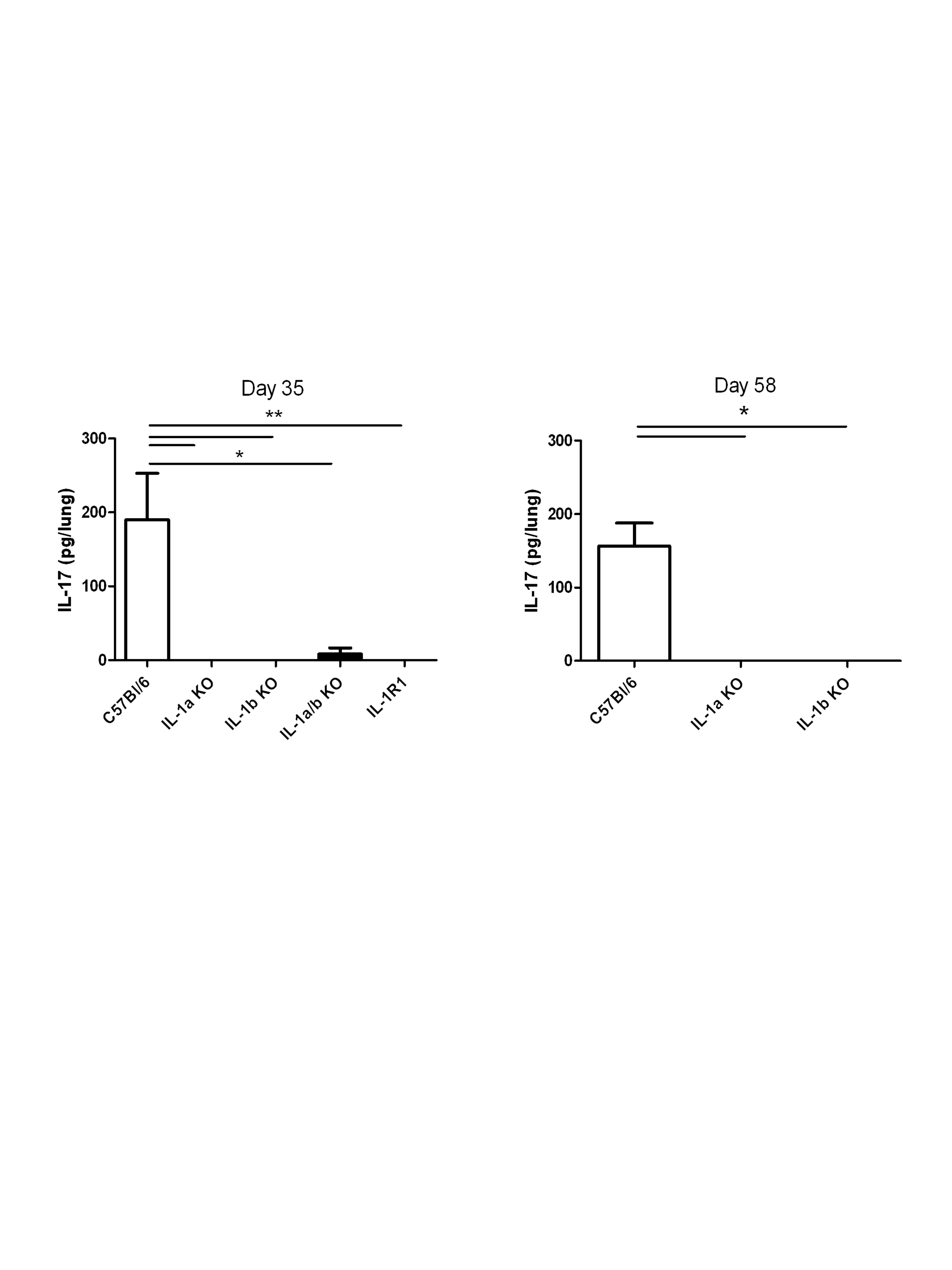

Supplement: Supplementary file 2 — Figure S2. Pulmonary levels of IL-17A in M. tuberculosis infected IL-1 deficient mice. IL-17A concentrations in lung homogenates of mice deficient for IL-1α, IL-1β, IL-1α plus IL-1β, or IL-1R1 and of wild-type mice were quantified by ELISA (Duoset R&D Systems) at 35 days after M. tuberculosis infection (left panel) or 56 days after M. tuberculosis infection (right panel). Results are expressed as mean ± SEM of cytokine levels reported to whole lungs, and are from n = 5 mice per group, except for two controls at day 56 (*P < 0.05; **P < 0.01; ***P < 0.001, as compared to wild-type controls). [file iid30001-0047-SD2.tif]
